# Tornado outbreak variability follows Taylor's power law of fluctuation scaling and increases dramatically with severity

**DOI:** 10.1038/ncomms10668

**Published:** 2016-02-29

**Authors:** Michael K. Tippett, Joel E. Cohen

**Affiliations:** 1 Department of Applied Physics and Applied Mathematics, Columbia University, New York, New York 10027, USA; 2 Center of Excellence for Climate Change Research, Department of Meteorology, King Abdulaziz University, Jeddah 21589, Saudi Arabia; 3 Laboratory of Populations, Rockefeller University, New York, New York 10065, USA; 4 The Earth Institute, Columbia University, New York, New York 10027, USA

## Abstract

Tornadoes cause loss of life and damage to property each year in the United States and around the world. The largest impacts come from ‘outbreaks' consisting of multiple tornadoes closely spaced in time. Here we find an upward trend in the annual mean number of tornadoes per US tornado outbreak for the period 1954–2014. Moreover, the variance of this quantity is increasing more than four times as fast as the mean. The mean and variance of the number of tornadoes per outbreak vary according to Taylor's power law of fluctuation scaling (TL), with parameters that are consistent with multiplicative growth. Tornado-related atmospheric proxies show similar power-law scaling and multiplicative growth. Path-length-integrated tornado outbreak intensity also follows TL, but with parameters consistent with sampling variability. The observed TL power-law scaling of outbreak severity means that extreme outbreaks are more frequent than would be expected if mean and variance were independent or linearly related.

## Abstract (web summary)

Tornadoes, which cause loss of life and damage to property worldwide, may occur in outbreaks of six or more in rapid succession. Here, the authors show that the annual mean number of tornadoes per US outbreak rose over the last 60 years, with the variance increasing four times faster than the mean.

## Graphical abstract

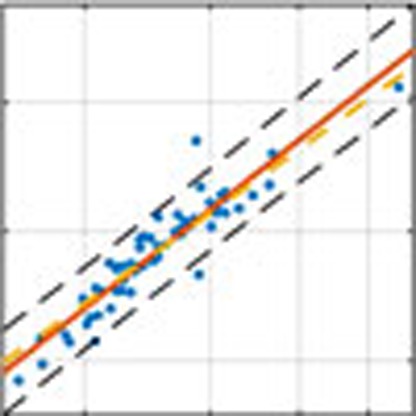

Hazardous convective weather (tornadoes, hail and damaging wind) associated with severe thunderstorms affects large portions of the United States. Tornadoes cause particularly intense damage. Over a recent 10-year period (2005–2014), tornadoes in the United States resulted in an average of 110 deaths per year and annual losses ranging from $500 million to $9.6 billion^1^. The largest societal impacts from tornadoes are from ‘outbreaks' in which multiple tornadoes occur in a single weather event. Tornado outbreaks across the eastern two-thirds of the United States were associated with 79% of all tornado fatalities over the period 1972–2010 (ref. 2) and are routinely responsible for billion-dollar loss events^3^.

Whether US tornado activity will change in the future remains uncertain. Climate change projections indicate that environments favourable to severe thunderstorms will be more frequent in a warmer climate^4^, and high-resolution convection-permitting numerical modelling indicates increased activity and year-to-year variability of March–May US tornado occurrence as measured by severe weather proxies derived from explicitly depicted storms^5^. However, to date, there is no upward trend in the number of reliably reported US tornadoes per year^6^. Interpretation of the US tornado report data requires some caution. For instance, the total number of US tornadoes reported each year has increased dramatically over the last half century, but most of that increase is due to more reports of weak tornadoes and is believed to reflect changing reporting practices and other non-meteorological factors rather than increased tornado occurrence^7^. The variability of reported tornado occurrence has increased over the last few decades with more tornadoes being reported on days when tornadoes are observed^8^,^9^. In addition, greater year-to-year variability in the number of tornadoes reported per year has been associated with consistent changes in the monthly averaged atmospheric environments favourable to tornado occurrence^10^. Likewise, US large-event severe thunderstorm losses and the frequency of the most extreme environments have increased^11^. Regional changes in the seasonality of tornado occurrence have also been reported^12^,^13^.

Despite their importance, relatively little is known about the current or projected statistics of tornado outbreak severity beyond their climatology^2^. Most studies have considered only the statistics of tornadoes occurring during a single day^14^ and have not considered outbreaks over multiple dates. Here we show that the annual mean number of tornadoes per outbreak increased during 1954–2014, and the annual variance increased more than four times faster than the mean. We show that the mean and variance of the number of tornadoes per outbreak are related by Taylor's power law of fluctuation scaling (TL)^15^,^16^ with parameters that are consistent with multiplicative growth^17^. TL scaling in tornado outbreak statistics was not previously known. Although power-law scaling is present in the probability distributions of various tornado characteristics^18^,^19^, the presence of power-law scaling in such probability distributions is neither necessary nor sufficient for TL scaling of the variance in relation to the mean (see also Supplementary Discussion for examples of TL scaling without power-law probability distributions). We also find that a tornado-related atmospheric proxy shows a similar power-law scaling and multiplicative growth. Path-length-integrated tornado outbreak intensity follows TL as well, but with parameters predicted by sampling variability^20^. The findings are similar when we restrict the analysis to the more recent period 1977–2014 and to more intense tornadoes. The observed TL scaling of outbreak severity means that extreme outbreaks are more frequent than would otherwise be expected if mean and variance were independent or linearly related.

## Results

### Data and sensitivity analyses

We use data from 1954 to 2014, which are generally considered reliable. Because of concerns regarding the data before 1977 (‘Methods' section, Supplementary Fig. 1), we repeat some analysis using the more recent period 1977–2014 to test the robustness of the results (Supplementary Figs 2–5). We exclude the weakest tornadoes from our analysis and denote the remaining tornadoes as F1+ tornadoes (‘Methods' section). We repeat some of the analysis restricted to more intense tornadoes (F2+; Supplementary Figs 7–10).

### Number of tornadoes per outbreak

**Figure 1:**
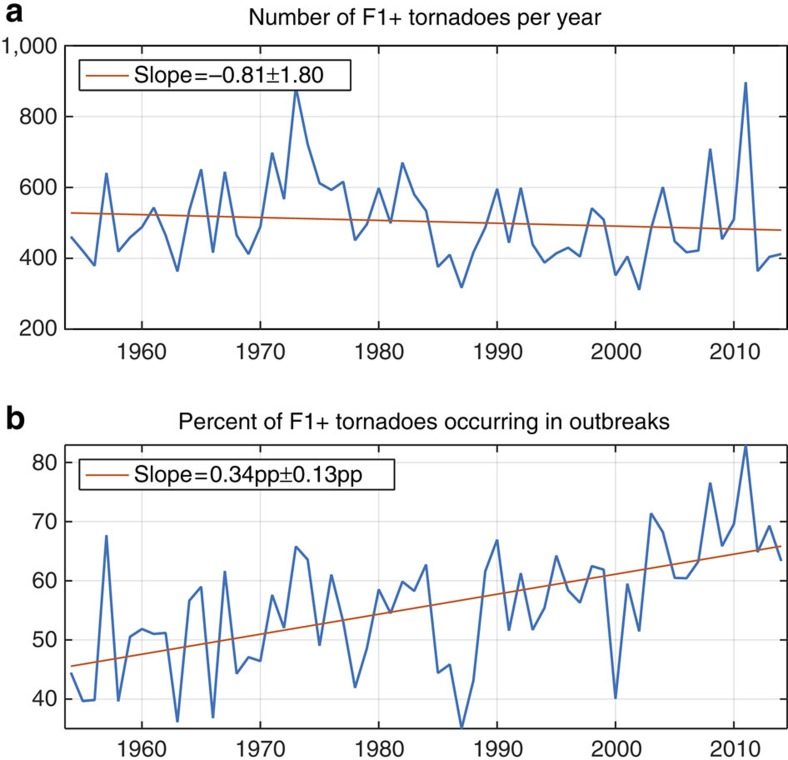
Time series of counts and clustering of F1+ tornadoes 1954–2014 in the contiguous US. (**a**) Number of F1+ tornadoes per year. The slope of the least-squares regression indicates that the number of F1+ tornadoes per year declined by 0.81 per year on average from 1954 to 2014 inclusive. This rate of decline is not statistically significantly different from 0 (no change). (**b**) Annual percentage of F1+ tornadoes occurring in outbreaks. The slope of the least-squares regression indicates that the percentage of F1+ tornadoes per year that occurred as part of outbreaks increased by 0.34 percentage points (pp) per year on average from 1954 to 2014 inclusive. This increase is statistically significantly greater than 0. In both **a** and **b**, ± intervals are 95% confidence intervals.

**Figure 2:**
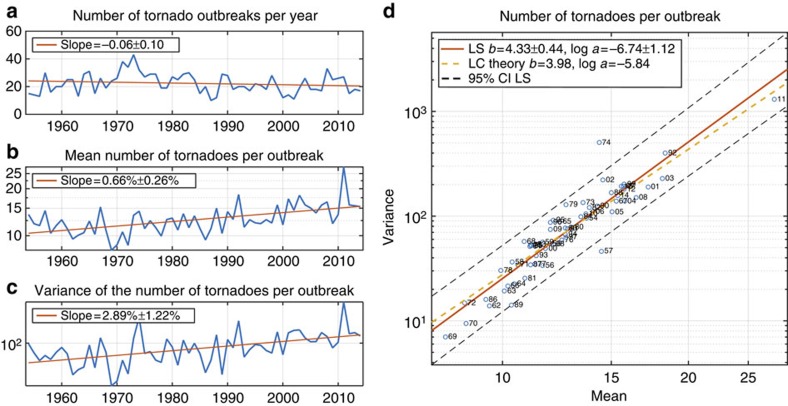
Numbers of F1+ tornadoes per outbreak 1954–2014. (**a**) Number of tornado outbreaks per year. The rate of decline is not statistically significantly different from 0 (no change). (**b**) Annual mean number of tornadoes per outbreak. Vertical axis is on logarithmic scale, so the rate of increase in the annual mean is expressed as a percentage per year. This rate of increase is statistically significantly greater than 0. (**c**) Annual variance of the number of tornadoes per outbreak. Vertical axis is on logarithmic scale, so the rate of increase in the annual mean is expressed as a percentage per year. This rate of increase is statistically significantly greater than 0. (**d**) Scatter plot of the annual mean number of tornadoes per outbreak versus the annual variance of the number of tornadoes per outbreak. Both axes are on logarithmic scale. The solid red line is the least-squares (LS) regression line (Taylor's power law of fluctuation scaling) and the dashed yellow line has the slope and intercept predicted by LC theory^17^. The two-digit number following the plotting symbol 'o' gives the calendar year in the second half of the twentieth century or first half of the twenty-first century. In all the panels, ± intervals are 95% confidence intervals.

The annual number of F1+ tornadoes shows no significant trend over the period 1954–2014 (Fig. 1a). Generally, trends have not been found in the number of severe tornadoes when severity is defined using the Fujita scale, but upward trends have been found when severity is defined using path length^18^. The percentage of F1+ tornadoes that occur in outbreaks (‘Methods' section) is increasing by 0.34 percentage points ±0.13 percentage points per year (Fig. 1b), consistent with upward trends in the proportion of tornadoes occurring on days with many tornadoes^8^,^9^. Here and in all results, ± intervals are 95% confidence intervals. The fraction of F1+ tornadoes that occur in outbreaks is less than one because not all F1+ tornadoes occur in outbreaks. During the period 1977–2014, the number of F1+ tornadoes also shows no significant trend, and the percentage of F1+ tornadoes occurring in outbreaks is also increasing, at a larger estimated rate (Supplementary Fig. 2). The US tornado reports show no statistically significant trend in the frequency of tornado outbreaks (Fig. 2a). Since the number of F1+ tornadoes and the number of outbreaks are not changing (on average, over time), the increasing percentage of F1+ tornadoes occurring in outbreaks means that the number of F1+ tornadoes per outbreak must be increasing, and indeed, the annual mean number of F1+ tornadoes per outbreak shows a significant upward trend (Fig. 2b). The annual mean number of tornadoes per outbreak is increasing by 0.66% ±0.26% per year, and the variance is increasing more than four times as fast, 2.89% ±1.22% per year (Fig. 2b,c) over the period 1954–2014. The growth rates are greater over the recent period 1977–2014, with similar ratio between the growth rates of mean and variance (Supplementary Fig. 3b,c).

The fact that the variance is increasing several times faster than the mean is especially noteworthy: it indicates a changing distribution in which the likelihood of extreme outbreaks is increasing faster than what the trend in mean alone would suggest. The coefficient of dispersion of a probability distribution with a positive mean is the ratio of its variance to its mean. Values greater than one (over-dispersion) indicate more clustering than a Poisson variable. For instance, European windstorms exhibit over-dispersion and serial clustering that increases with intensity^21^ with implications for the return intervals of rare events^22^. Taylor's law (TL) relates the mean and variance of a probability distribution by


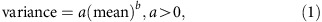


where *a* and *b* are constants^15^,^16^. A value of *b>*1 indicates that the coefficient of dispersion increases with the mean. The annual mean and annual variance of the number of tornadoes per outbreak approximately satisfy TL with *b*=4.3±0.44 and log *a*=−6.74±1.12 (Fig. 2d); consistent values are seen over the period 1977–2014 (Supplementary Fig. 3d). (Throughout log is the natural logarithm.) The value of *b* here is remarkable since in most ecological applications, the TL exponent seldom exceeds 2. The TL exponent can be greater than 2 for lognormal distributions with changing parameters (Supplementary Discussion and Supplementary Fig. 11). The TL scaling of tornado outbreak severity reveals a remarkably regular relation between annual mean and annual variance that extends over the full range of the data, even for years like 2011 which are extreme in mean and variance. The data from 1974 deviate most from TL scaling, with the excessive variance reflecting the 3–4 April ‘Super Outbreak.'

The upward trend in the number of tornadoes per outbreak provides an interpretation for the observed TL scaling since TL scaling arises in models of stochastic multiplicative growth^17^. In such models, the quantity *N*(*t*+1) at time *t*+1 is related to its previous value *N*(*t*) by


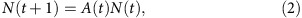


where *A*(*t*) is the random multiplicative factor by which *N*(*t*) grows or declines from one time to the next. Here *N*(*t*) is the annual average number of tornadoes per outbreak, and each integer value of *t* represents one calendar year. The Lewontin–Cohen (LC) model for stochastic multiplicative growth assumes that the *A*(*t*) are independently and identically distributed for all *t*≥0 with finite mean *M*>0 and finite variance *V* . If *M*≠1, *N*(*t*) follows TL asymptotically with^17^


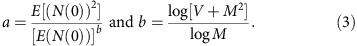


Here we estimate (‘Methods' section) *M*=1.03 and *V*=0.068, which leads to TL parameters *b*=3.98 and log(*a*)= −5.84. Both values are consistent with the least-squares (LS) estimates of the corresponding parameters of TL (Fig. 2d). The LS estimates are also consistent with the values from LC theory during 1977–2014 (Supplementary Fig. 3d). 95% confidence intervals for *M* and *V* show that the hypothesis of no growth (*M*=1) under which equation (3) is not valid cannot be rejected (Supplementary Table 1). The Supplementary Discussion provides additional description of how the LC model leads to TL scaling with exponent approximately 4.

### Fujita-kilometers per outbreak

**Figure 3:**
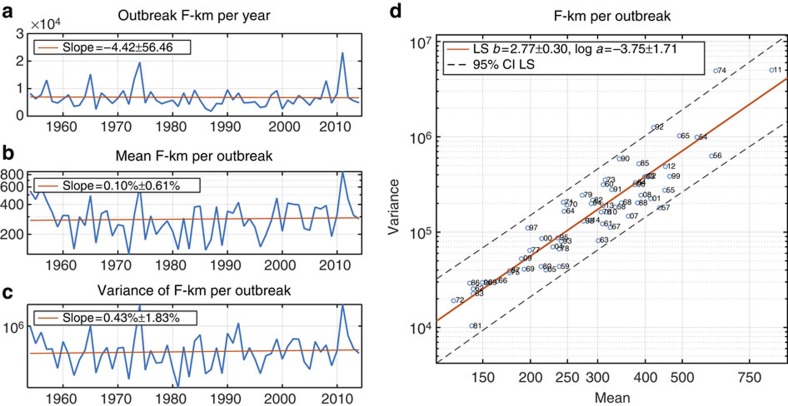
F-km per outbreak 1954–2014. (**a**) Total outbreak F-km per year. The rate of decline is not statistically significantly different from 0 (no change) among F1+ tornadoes. (**b**) Annual mean F-km per outbreak. Vertical axis is on logarithmic scale, so the rate of increase in the annual mean is expressed as a percentage per year. This rate of increase is not statistically significantly greater than 0. (**c**) Annual variance of F-km per outbreak. Vertical axis is on logarithmic scale, so the rate of increase in the annual mean is expressed as a percentage per year. This rate of increase is not statistically significantly greater than 0. (**d**) Scatter plot of the annual mean of F-km per outbreak versus the annual variance of F-km per outbreak. Both axes are on logarithmic scale. The solid red line is the least-squares (LS) regression line (Taylor's power law of fluctuation scaling). The two-digit number following the plotting symbol 'o' gives the calendar year in the second half of the twentieth century or first half of the twenty-first century. In all the panels, ± intervals are 95% confidence intervals.

Another measure of outbreak severity is Fujita-kilometers (ref. 2; F-km) which is the sum (over all tornadoes in an outbreak) of each tornado's path length in kilometers multiplied by its Fujita or Enhanced Fujita rating (‘Methods' section). Annual totals of outbreak F-km, mean number of F-km per outbreak and the variance of F-km per outbreak do not show significant trends over the period 1954–2014 (Fig. 3a–c). The mean number of F-km per outbreak and the variance of F-km per outbreak show marginally significant trends over the recent period 1977–2014 (Supplementary Fig. 4a–c). The TL parameters relating the mean and variance of F-km per outbreak are *b*=2.77±0.30 and log *a*=−3.75±1.71 (Fig. 3d). The lack of robust trends means that LC theory is not appropriate to explain the TL scaling of F-km. However, TL scaling also arises from the sampling of stationary skewed distributions^20^. For a distribution with mean *m,* variance *v,* skewness 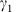 and coefficient of variation *CV,* theory^20^ predicts


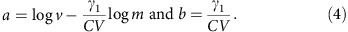


Here, excluding two outlier outbreaks from the calculation of the distribution parameters (‘Methods' section, Supplementary Fig. 5), equation (4) gives *b*=2.71 and log *a*=−3.05, both of which are consistent with the LS estimates of the TL parameters for F-km per outbreak. (We use ‘outlier' to indicate values far from other observations, not to suggest that the unusual values are the result of measurement error.) Therefore TL scaling of F-km per outbreak could be explained by sampling variability.

### A tornado environment proxy

**Figure 4:**
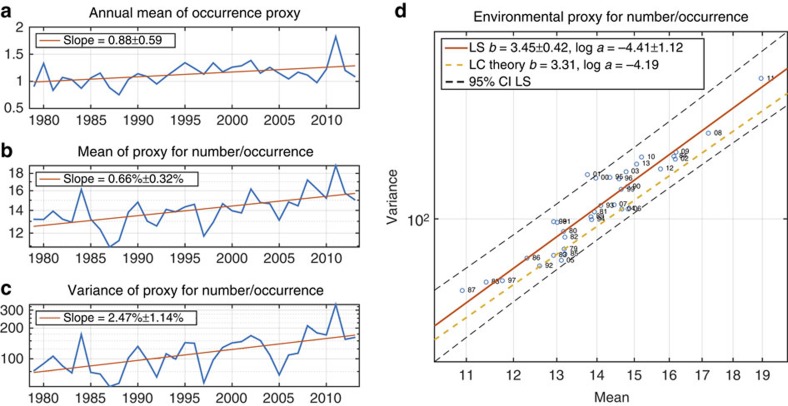
Environmental proxies 1979–2013. (**a**) Annual mean of occurrence proxy in per cent. The rate of increase is statistically significantly greater than 0. (**b**) Annual mean of the environmental proxy for number of tornadoes per occurrence. Vertical axis is on logarithmic scale, so the rate of increase in the annual mean is expressed as a percentage per year. This rate of increase is statistically significantly greater than 0. (**c**) Annual variance of the environmental proxy for number of tornadoes per occurrence. Vertical axis is on logarithmic scale, so the rate of increase in the annual mean is expressed as a percentage per year. This rate of increase is statistically significantly greater than 0. (**d**) Scatter plot of the annual mean of proxy for number of tornadoes per occurrence versus the annual variance of proxy for number of tornadoes per occurrence. Both axes are on logarithmic scale. The solid red line is the least-squares (LS) regression line and the dashed yellow line has slope and intercept predicted by LC theory^17^. The two-digit number following the plotting symbol 'o' gives the calendar year in the second half of the twentieth century or first half of the twenty-first century. In all the panels, ± intervals are 95% confidence intervals.

A reasonable concern is that the findings here represent properties of the tornado report database that are not meteorological in origin, especially since other prominent features of the tornado report database are not meteorological in origin^7^. Environmental proxies for tornado occurrence and number of tornadoes per occurrence provide an independent, albeit imperfect, measure of tornado activity for the period 1979–2013 (‘Methods' section). At a minimum, the environmental proxies provide information about the frequency and severity of environments favourable to tornado occurrence. The correlation between the annual average number of tornadoes per outbreak and the proxy for number of tornadoes per occurrence is 0.56 (Supplementary Fig. 6a). This correlation falls to 0.34, still significant at the 95% level, when the data from 2011 are excluded. Applying a 5-year moving average to the data highlights their common trends and increases the correlation to 0.88 (Supplementary Fig. 6b). The annual mean of the occurrence proxy, a surrogate for number of tornadoes per year, shows a marginally significant upward trend (Fig. 4a). The annual mean and annual variance of the proxy for number of tornadoes per occurrence show upward trends of 0.63±0.30% and 2.43±1.12%, respectively (Fig. 4b,c), values strikingly similar to those for number of tornadoes per outbreak (Fig. 2b,c). Moreover, the TL parameters of the proxy for number of tornadoes per occurrence are 3.54±0.42 and log *a*=−5.87±1.43, which are consistent with the LC multiplicative growth theory estimates for the proxy (Fig. 4d) and quite similar to those for the number of tornadoes per outbreak. Extreme environments associated with tornado occurrence display the TL scaling and multiplicative growth similar to those of the number of tornadoes per outbreak. This similarity plausibly suggests that the changes in the number of tornadoes per outbreak reflect changes in the physical environment.

### Sensitivity to outbreak definition

Another concern is that the results are sensitive to the details of the outbreak definition. We assess the robustness of the results to the E/F1 threshold by repeating the analysis with tornadoes rated E/F2 and higher, denoted F2+ (Supplementary Figs 7–10). We use the period 1977–2014 because the annual number of F2+ tornadoes display a substantial decrease (not shown) around the 1970s that is likely related to the introduction of the F-scale. Overall the F2+ results are remarkably similar to the F1+ ones. The annual number of F2+ tornadoes has an insignificantly negative trend during 1977–2014 (Supplementary Fig. 7a), and the percentage of F2+ tornadoes occurring in F2+ outbreaks has a significant positive trend (Supplementary Fig. 7b). Although the number of F2+ outbreaks shows no significant trend, the mean number of tornadoes per F2+ outbreak and its variance both have significant upward trends (Supplementary Fig. 8a–c). The TL exponent for number of tornadoes per F2+ outbreak is 3.65 and is consistent with LC theory (Supplementary Fig. 8d). Annual totals of F2+ outbreak F-km have no significant trend (Supplementary Fig. 9a). Mean F-km per F2+ outbreak does have a significant upward trend, but variance does not (Supplementary Fig. 9b,c). The TL scaling of F2+ outbreak F-km (Supplementary Fig. 9d) is consistent with sampling variability when 2011 is excluded (Supplementary Fig. 10).

## Discussion

These findings have important implications for tornado risk in the United States and perhaps elsewhere, though we have examined only the US data. First, the number of tornadoes per outbreak is increasing. However, there is less evidence that F-km per outbreak are increasing. Both the number of tornadoes per outbreak and F-km per outbreak follow TL, which relates mean and variance. We find that TL scaling for the number of tornadoes per outbreak is compatible with multiplicative growth, and that TL scaling for F-km per outbreak could be due to sampling variability. Finally, the key implication of TL scaling is that both number of tornadoes per outbreak and F-km per outbreak exhibit extreme over-dispersion which increases with mean. When the average tornado outbreak severity gets worse, the high extreme of severity rises even faster and the low extreme falls even faster, by either measure of severity.

## Methods

### Outbreak data

Tornado reports come from the NOAA Storm Prediction Center (http://www.spc.noaa.gov/wcm/#data). There are no reports from either Alaska or Hawaii; one report from Puerto Rico is excluded. Tornadoes are rated by estimated or reported damage using the Fujita (F) scale, introduced in the mid-1970s, and since March 2007, the enhanced Fujita (EF) scale, with 0 being the weakest and 5 the strongest. Only reports of tornadoes rated F/EF1 or greater are used and are denoted as F1+. To compute outbreak statistics, tornado reports are first sorted in chronological order taking into account the time zone. Outbreaks are sequences of 6 or more F1+ tornadoes (regardless of location in the contiguous United States) whose successive start times are separated by no more than 6 h (ref. 2). A total 1,361 outbreaks are found over the period 1954–2014. Outbreaks spanning more than 1 day are possible with this definition. The median outbreak duration is about 8 h, and 95% of the outbreaks last less than 24 h. Additional climatological features can be found in ref. 2 The number of tornadoes and F-km are computed for each outbreak. Then the annual mean and variance of two outbreak severity measures, number of tornadoes per outbreak and F-km per outbreak, are calculated. The distribution of outbreak tornadoes by F/EF-scale shows considerable variation before 1977 (Supplementary Fig. 1) and may well reflect lower reliability in the earlier period^23^ before damage surveys were a routine part of the rating procedure. Therefore, we repeat some of our analysis using the recent period 1977–2014 (Supplementary Figs 2,3).

The same outbreak calculation procedure is repeated but considering only reports of tornadoes rated F/EF2 or greater, denoted as F2+. These outbreak events are referred to as F2+ outbreaks (Supplementary Figs 7–10). Only F2+ tornadoes are used to calculate tornado numbers and F-km of F2+ outbreaks.

### Trends

All trends and 95% confidence intervals are assessed using linear regression and ordinary least squares, assuming approximately normal distributions of residuals. The growth rates of the annual mean and variance of the outbreak severity measures in Fig. 2b,c, Fig. 3b,c and Supplementary Figs 3b,c; 4b,c; 8b,c and 9b,c are computed by assuming exponential growth and fitting a linear trend to the logarithms of the data. All the other trends are fitted using untransformed data.

### TL parameters

The TL parameters in Figs 2d, 3d and 4d and Supplementary Figs 3d, 4d, 8d and 9d and 95% confidence intervals are estimated using ordinary least-squares regression with the logarithms of the mean and variance.

### TL parameters implied by LC theory

The growth factor in the LC model is computed from *A*(*t*)=*N*(*t*+1)/*N*(*t*), where *N*(*t*) is the average number of tornadoes per outbreak in calendar year *t*. The 95% confidence intervals for the mean and variance of *A*(*t*) are computed from 10,000 bootstrap samples and reported in Supplementary Table 1. The mean and variance of *A*(*t*) are used to compute a prediction of the slope *b* using equation (3). The TL parameter *a* is estimated from equation (1) evaluated at the initial year, either 1954 or 1977.

### TL parameters implied by sampling variability

There are 1,361 cases of outbreak F-km, and their distribution is highly right-skewed (Supplementary Figs 5a and 10a). The F-km values for the 1974 Super Outbreak and the 25–28 April 2011 tornado outbreak are more than 26 standard deviations above the mean of the data on an arithmetic scale and more than six standard deviations above the mean of the log-transformed data, when means and standard deviations are calculated after withholding the two extreme values. These outliers (values that are far from other observations) have a substantial impact on the estimates of the mean, variance and skewness of the F-km distribution. Despite their rarity, about 86% (1−(1−2/1,361)^1361^) of the bootstrap samples will contain one or both of these two events. The presence of the outliers results in bimodal distributions of the TL slope and intercept estimates (Supplementary Figs 5b,c and 10b,c) computed from equation (4), depending on whether or not the outlier values are in the particular bootstrap sample. Removal of the outliers results in unimodal distributions, whose ranges are consistent with the least-squares estimates of the TL slope and intercept for F-km (Supplementary Figs 5d,e and 10d,e).

### Environmental proxies

We use two environmental proxies: one for tornado occurrence and one for the number of tornadoes. Tornadoes are often associated with elevated values of convective available potential energy (CAPE; J kg^−1^) and a measure of vertical wind shear, called storm relative helicity (SRH; m^2^ s^−2^). Here 0–180 hPa CAPE and 0–3,000 m SRH data are taken from the North American Regional Reanalysis^24^. The data are interpolated to a 1 × 1 degree latitude–longitude grid over the continental United States and daily averages are computed. The environmental proxy for tornado occurrence is defined using the energy-helicity index^25^ (EHI), which is the product of CAPE and SRH divided by 160,000 J kg^−1^ m^2^ s^−2^. Values of EHI greater than one indicate the potential for supercell thunderstorms and tornadoes^26^, and accordingly we take our proxy for tornado occurrence to be the condition that EHI is greater than 1. Selection of an environmental proxy for the number of tornadoes is more challenging. The only example of a proxy calibrated to predict the number of tornadoes from the surrounding environment uses monthly averaged environment and monthly number of tornadoes^27^,^28^, and therefore is not suited for outbreaks, which last much less than a month. However, previous research does provide some indications for the functional form of such a proxy. For instance, on subdaily timescales the likelihood of significant severe weather is nearly twice as sensitive to vertical wind shear as to CAPE^29^,^30^. This sensitivity would argue for a proxy based on the product of CAPE and the square of vertical wind shear or equivalently the product of the square root of CAPE and vertical wind shear^31^. Likewise, proxies for the monthly number of tornadoes contain SRH with an exponent ranging from 1.89 to 4.36 depending on region^27^,^28^. The supercell composite parameter^32^ is used in weather forecasting and is the product of CAPE, SRH and vertical shear, again indicating a scaling of severe weather with the square of vertical wind shear measures. On the basis of this evidence, we take as the proxy for number of tornadoes


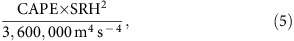


which differs from the EHI in that the square of SRH appears. The normalizing factor is chosen to match overall annual outbreak numbers. The proxy for the number of tornadoes per occurrence is therefore the mean of equation (5) conditional on the occurrence proxy of EHI being greater than one.

## Additional information

**How to cite this article:** Tippett, M. K. & Cohen J. E. Tornado outbreak variability follows Taylor's power law of fluctuation scaling and increases dramatically with severity. *Nat. Commun.* 7:10668 doi: 10.1038/ncomms10668 (2016).

## Supplementary Material

Supplementary Information Supplementary Figures 1-11, Supplementary Table 1, Supplementary Discussion and Supplementary References.
