# Supplementary material for: Tornado outbreak variability follows Taylor's power law of fluctuation scaling and increases dramatically with severity
Source: Nat Commun. 2016 Feb 29;7:10668. doi: 10.1038/ncomms10668 (PMC4773447; doi:10.1038/ncomms10668)
Supplement: Supplementary Information — Supplementary Figures 1-11, Supplementary Table 1, Supplementary Discussion and Supplementary References. [file ncomms10668-s1.pdf]

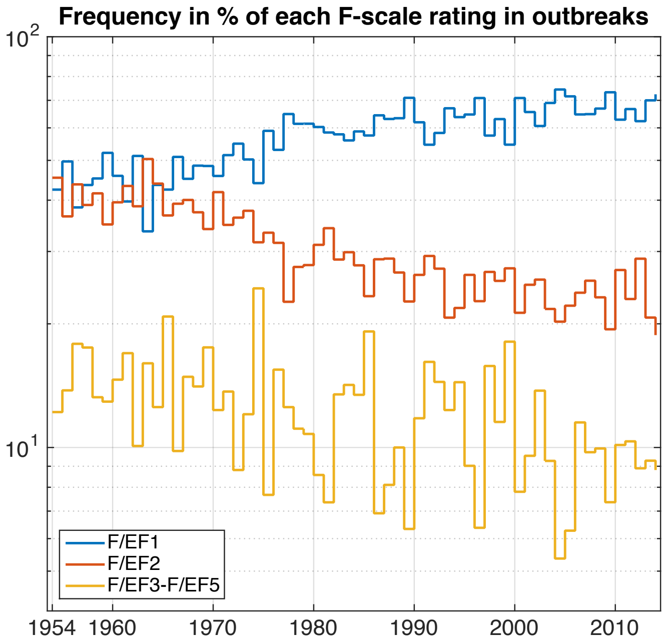

Supplementary Figure 1 | Distribution of outbreak tornadoes by F/EF-scale rating. Frequency (in percent per year) of F/EF1 (blue), F/EF2 (red) and F/EF3-F/EF5 (yellow) rated tornadoes in tornado outbreaks. Percentages sum to 100% in each year.

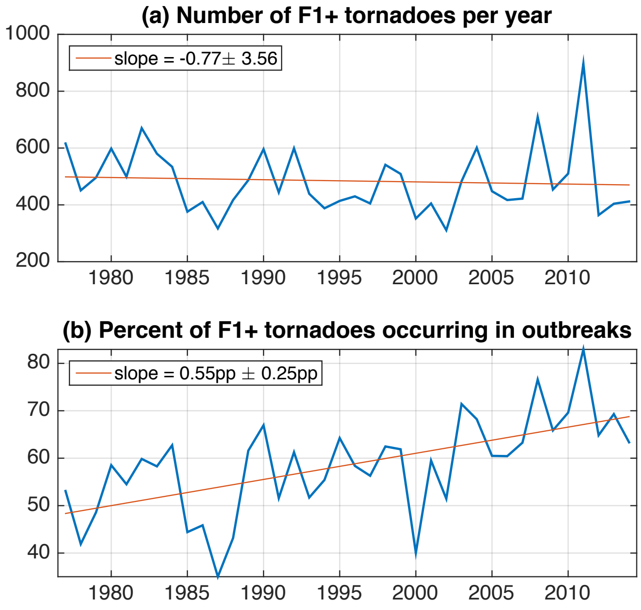

Supplementary Figure 2 | Time series of counts and clustering of F1+ tornadoes 1977-2014 in the contiguous US. (**a**) Number of F1+ tornadoes per year. The slope of the least-squares regression indicates that the number of F1+ tornadoes per year declined by 0.77 per year on average from 1977 to 2014 inclusive. This rate of decline is not statistically significantly different from 0 (no change). (**b**) Annual percentage of F1+ tornadoes occurring in outbreaks. The slope of the least-squares regression indicates that the percentage of F1+ tornadoes per year that occurred as part of outbreaks increased by 0.55 percentage points (pp) per year on average from 1977 to 2014 inclusive. This increase is statistically significantly greater than 0. In both (**a**) and (**b**), ± intervals are 95% confidence intervals.

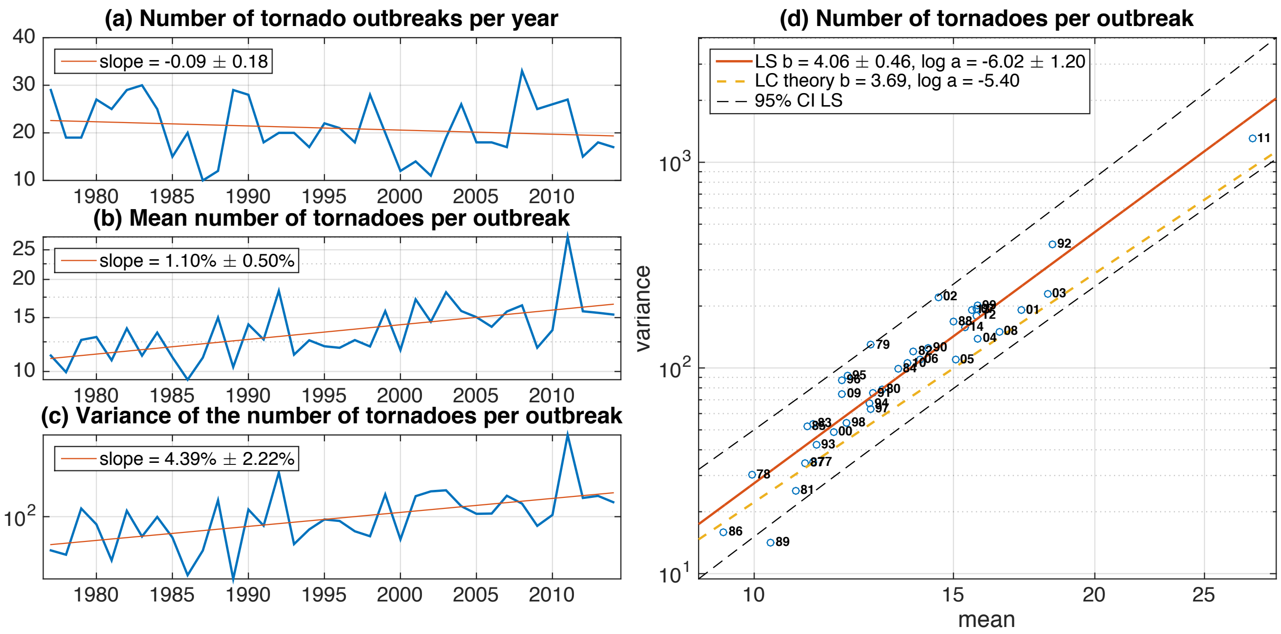

Supplementary Figure 3 | Numbers of F1+ tornadoes per outbreak 1977-2014. (**a**) Number of tornado outbreaks per year. The rate of decline is not statistically significantly different from 0 (no change). (**b**) Annual mean number of tornadoes per outbreak. Vertical axis is on logarithmic scale, so the rate of increase in the annual mean is expressed as a percentage per year. This rate of increase is statistically significantly greater than 0. (**c**) Annual variance of the number of tornadoes per outbreak. Vertical axis is on logarithmic scale, so the rate of increase in the annual mean is expressed as a percentage per year. This rate of increase is statistically significantly greater than 0. (**d**) Scatter plot of the annual mean number of tornadoes per outbreak versus the annual variance of the number of tornadoes per outbreak. Both axes are on logarithmic scale. The solid red line is the least-squares (LS) regression line (Taylor's power law of fluctuation scaling) and the dashed yellow line has the slope and intercept predicted by LC theory[1]. The two-digit number following the plotting symbol 'o' gives the calendar year in the second half of the 20th century or first half of the 21st century. In all panels, ± intervals are 95% confidence intervals.

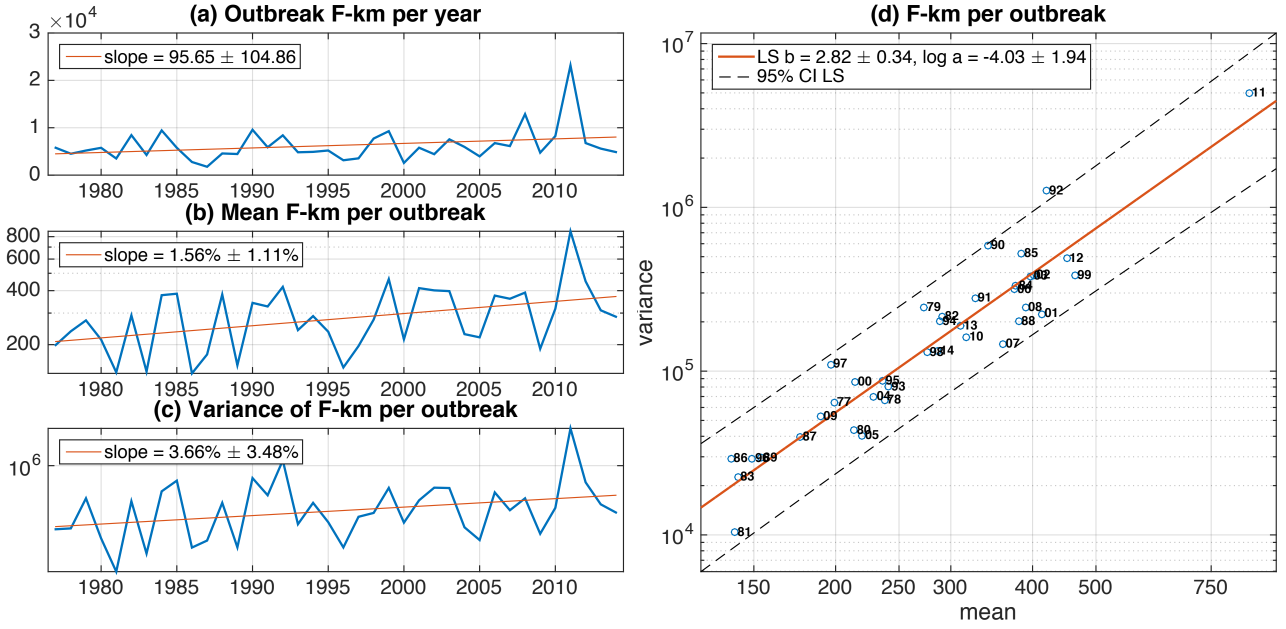

Supplementary Figure 4 | F-km per outbreak 1977-2014. (**a**) Total outbreak F-km per year. The rate of increase is not statistically significantly different from 0 (no change) among F1+ tornadoes. (**b**) Annual mean F-km per outbreak. Vertical axis is on logarithmic scale, so the rate of increase in the annual mean is expressed as a percentage per year. This rate of increase is statistically significantly greater than 0. (**c**) Annual variance of F-km per outbreak. Vertical axis is on logarithmic scale, so the rate of increase in the annual variance is expressed as a percentage per year. This rate of increase is statistically significantly greater than 0. (**d**) Scatter plot of the annual mean of F-km per outbreak versus the annual variance of F-km per outbreak. Both axes are on logarithmic scale. The solid red line is the least-squares (LS) regression line (Taylor's power law of fluctuation scaling). The two-digit number following the plotting symbol 'o' gives the calendar year in the second half of the 20th century or first half of the 21st century. In all panels, ± intervals are 95% confidence intervals.

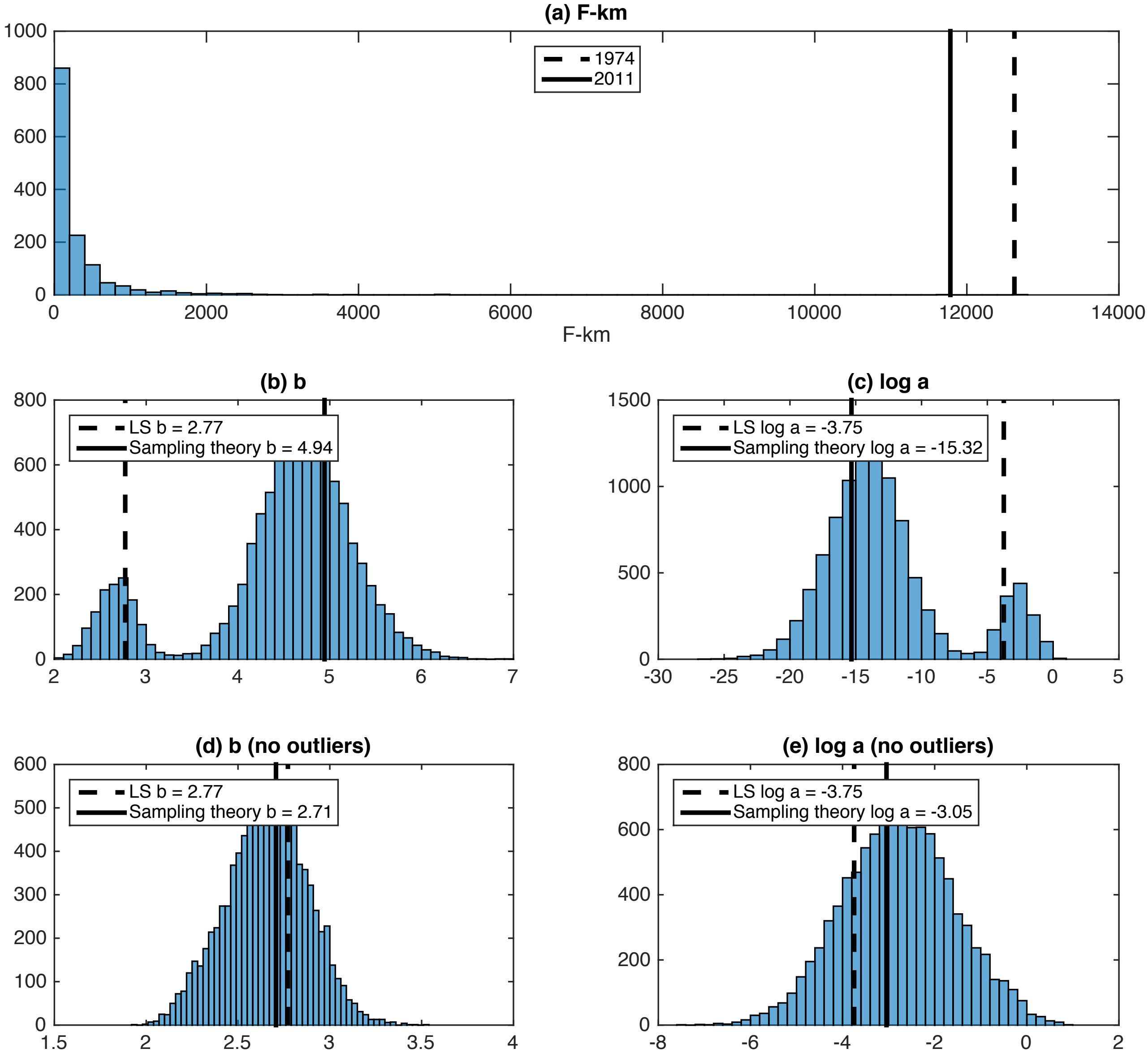

Supplementary Figure 5 | Distribution of F-km and sampling theory estimates of TL parameters for outbreaks 1954-2014. (**a**) Frequency distribution of F-km including the extreme values of 1974 and 2011. (**b-c**) Histogram of the TL parameters estimated from 10,000 bootstrap samples. (**d-e**) Histogram of the TL parameters estimated from 10,000 bootstrap samples excluding the extreme values of 1974 and 2011. The vertical axis is the frequency of occurrence among the 10,000 bootstrap samples.

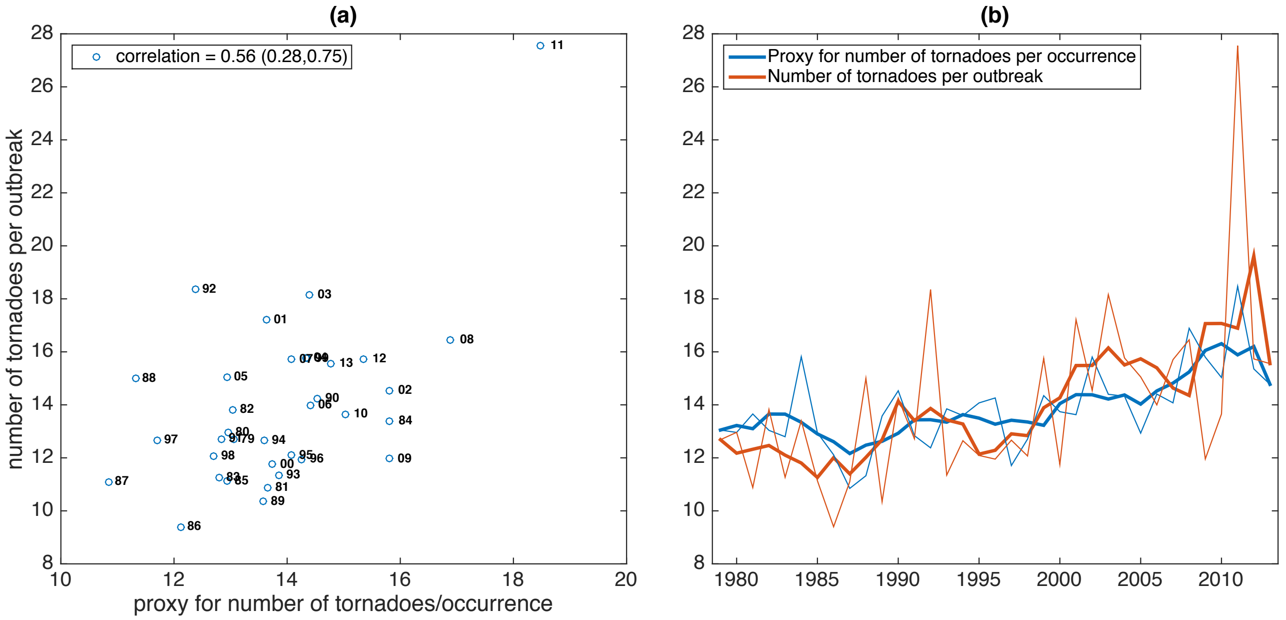

Supplementary Figure 6 | Environmental proxy. (**a**) Scatter plot of the annual mean of number of F1+ tornadoes per outbreak and the annual average of the environmental proxy for number of tornadoes per occurrence for 1979-2013. Two-digit numbers indicate year. The linear correlation is 0.56. (**b**) Annual (thin lines) and 5-year moving average (thick lines) time series of the mean of number of tornadoes per outbreak and the average of the environmental proxy for number of tornadoes per occurrence for 1979-2013. The correlation of the 5-year moving averages is 0.88.

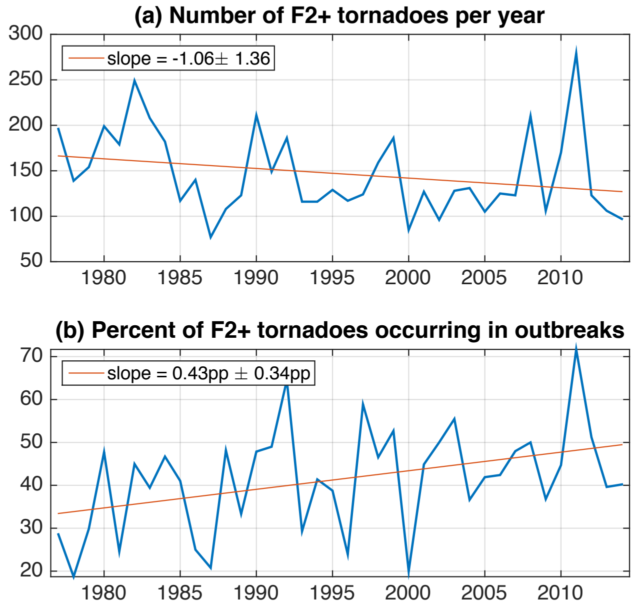

Supplementary Figure 7 | Time series of counts and clustering of F2+ tornadoes 1977-2014 in the contiguous US. (**a**) Number of F2+ tornadoes per year. The slope of the least-squares regression indicates that the number of F2+ tornadoes per year declined by 1.06 per year on average from 1977 to 2014 inclusive. The rate of decrease is not statistically significantly different from 0 (no change). (**b**) Annual percentage of F2+ tornadoes occurring in F2+ outbreaks. The slope of the least-squares regression indicates that the percentage of F1+ tornadoes per year occurring in outbreaks increased by 0.43 percentage points (pp) per year on average from 1977 to 2014 inclusive. This rate of increase is statistically significantly greater than 0. In both (**a**) and (**b**), ± intervals are 95% confidence intervals.

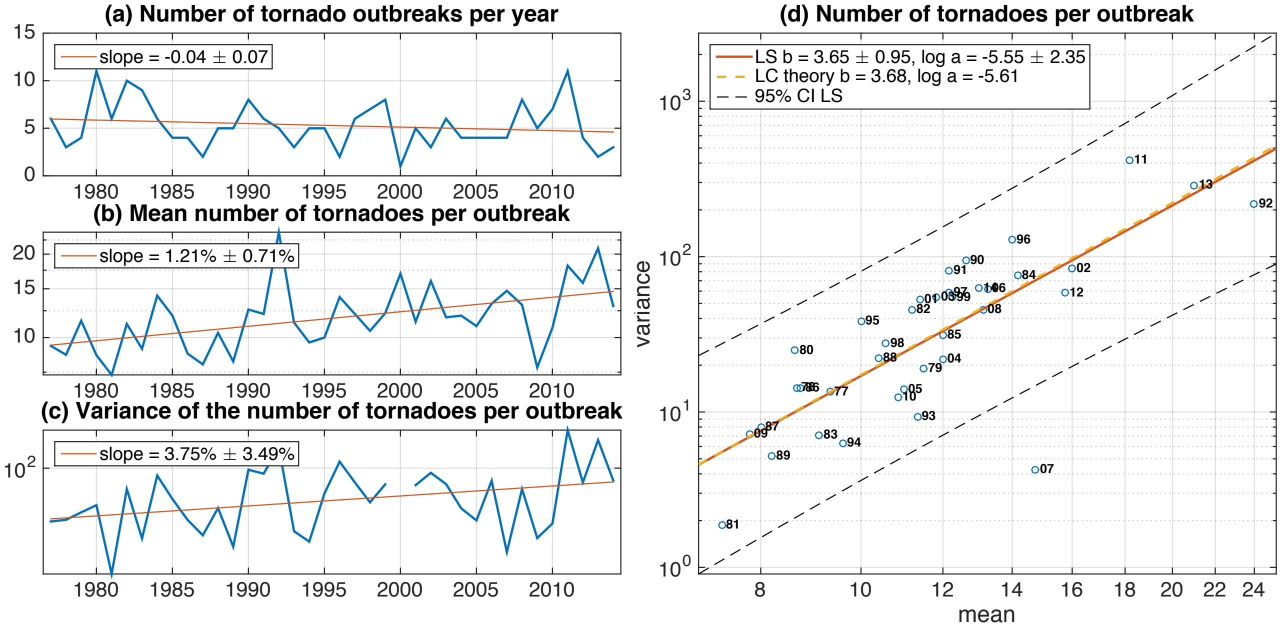

Supplementary Figure 8 | Numbers of F2+ tornadoes per F2+ outbreak 1977-2014. (**a**) Number of F2+ outbreaks per year. The rate of decline is not statistically significantly different from 0 (no change). (**b**) Annual mean number of tornadoes per F2+ outbreak. Vertical axis is on logarithmic scale, so the rate of increase in the annual mean is expressed as a percentage per year. This rate of increase is statistically significantly greater than 0. (**c**) Annual variance of the number of tornadoes per F2+ outbreak. Vertical axis is on logarithmic scale, so the rate of increase in the annual mean is expressed as a percentage per year. This rate of increase is statistically significantly greater than 0. (**d**) Scatter plot of the annual mean number of tornadoes per F2+ outbreak versus the annual variance of the number of tornadoes per F2+ outbreak. Both axes are on logarithmic scale. The solid red line is the least-squares (LS) regression line (Taylor's power law of fluctuation scaling) and the dashed yellow line has the slope and intercept predicted by LC theory[1]. The two-digit number following the plotting symbol 'o' gives the calendar year in the second half of the 20th century or first half of the 21st century. In all panels, ± intervals are 95% confidence intervals. There is one F2+ outbreak in 2000, and the variance of the number of tornadoes per F2+ outbreak in 2000 is not defined.

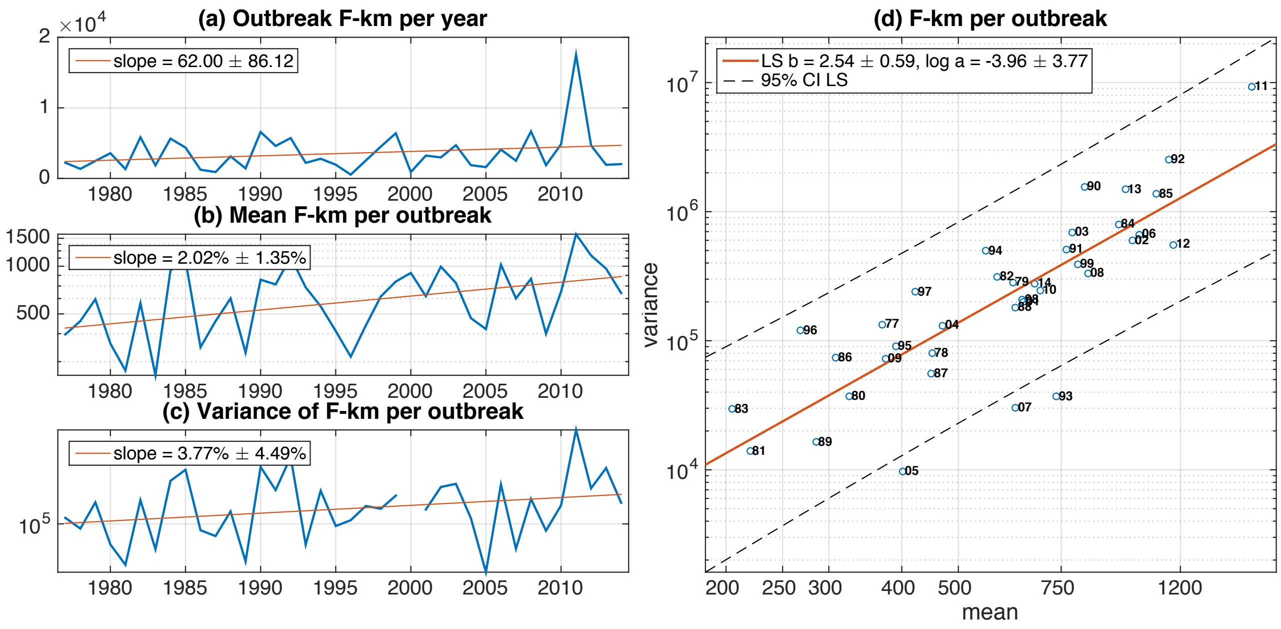

Supplementary Figure 9 | F-km per F2+ outbreak 1977-2014. (**a**) Total outbreak F-km per year. The rate of increase is not statistically significantly different from 0 (no change) among F2+ tornadoes. (**b**) Annual mean F-km per F2+ outbreak. Vertical axis is on logarithmic scale, so the rate of increase in the annual mean is expressed as a percentage per year. This rate of increase is statistically significantly greater than 0. (**c**) Annual variance of F-km per outbreak. Vertical axis is on logarithmic scale so the rate of increase in the annual mean is expressed as a percentage per year. This rate of increase is not statistically significantly greater than 0. (**d**) Scatter plot of the annual mean of F-km per F2+ outbreak versus the annual variance of F-km per F2+ outbreak. Both axes are on logarithmic scale. The solid red line is the least-squares (LS) regression line (Taylor's power law of fluctuation scaling). The two-digit number following the plotting symbol 'o' gives the calendar year in the second half of the 20th century or first half of the 21st century. In all panels, ± intervals are 95% confidence intervals. There is one F2+ outbreak in 2000, and the log variance of the number of tornadoes per F2+ outbreak in 2000 is not defined.

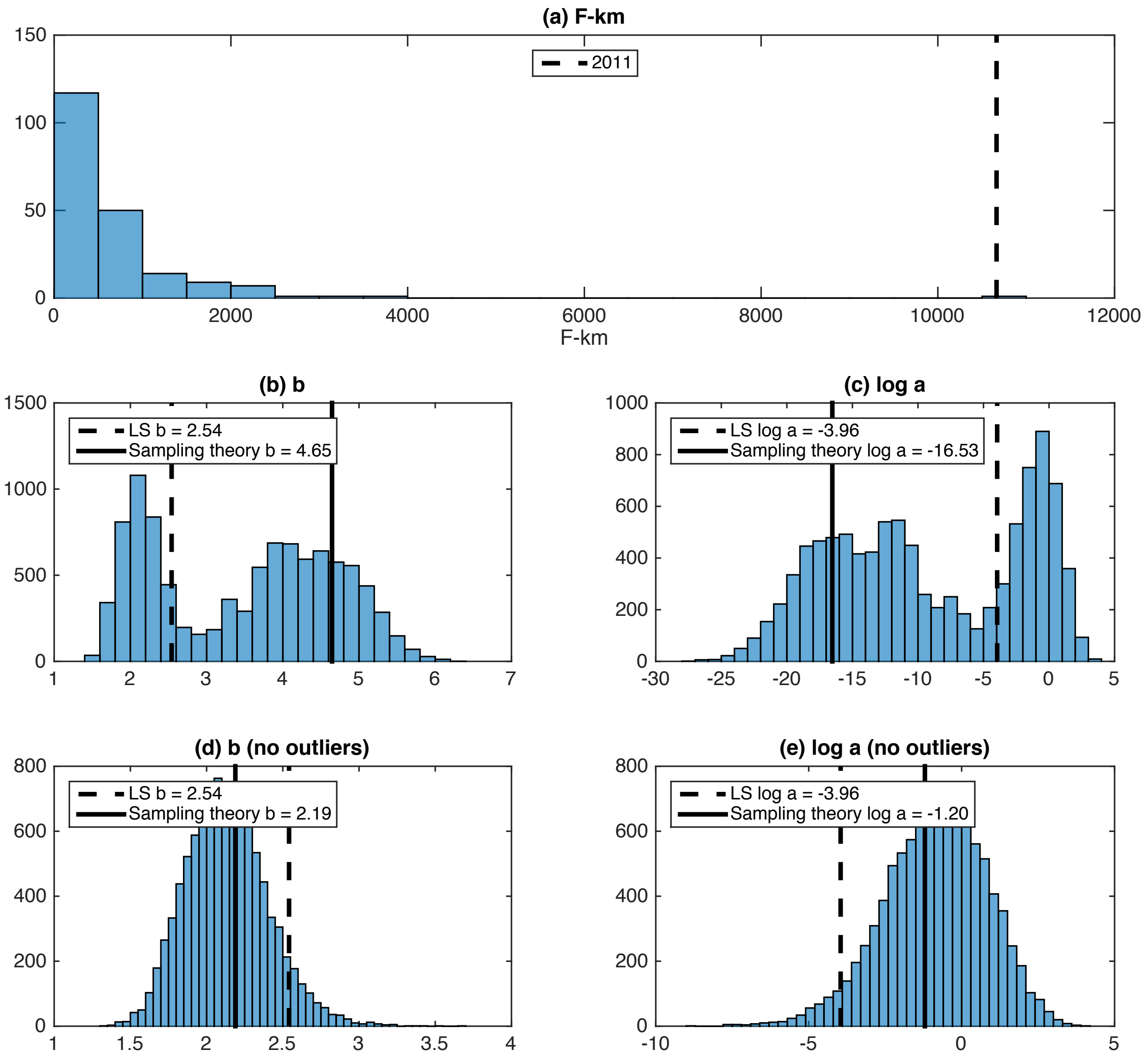

Supplementary Figure 10 | Distribution of F-km and sampling theory estimates of TL parameters for F2+ outbreaks 1977-2014. (**a**) Frequency distribution of F-km including the extreme value of 2011. (**b-c**) Histogram of the TL parameters estimated from 10,000 bootstrap samples. (**d-e**) Histogram of the TL parameters estimated from 10,000 bootstrap samples excluding the extreme value of 2011.

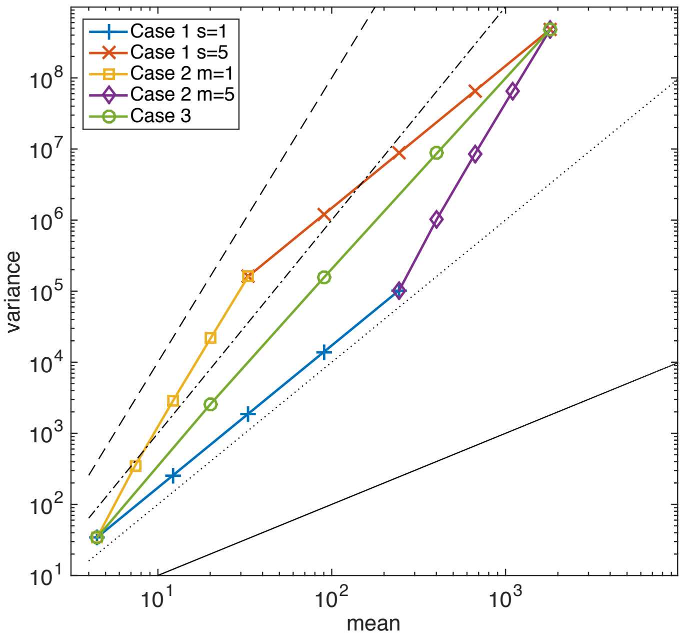

Supplementary Figure 11 | Taylor's law scaling for lognormal distributions with changing parameters. The thin black diagonal lines that pass through the origin have slopes 1 (solid line), 2 (dotted line), 3 (dash-dotted line), and 4 (dashed line), reading from the bottom to the top. These are guidelines for the eye. The thicker lines join markers which plot values of $(x, y) = (\mu(m, s^2), \sigma^2(m,s^2))$. Case 1 is illustrated by the two sides of the parallelogram with slope exactly 2, where $m = 1, 2, 3, 4, 5$ and $s^2 = 1$ (blue + markers, along the bottom of the parallelogram) and $m = 1, 2, 3, 4, 5$ and $s^2 = 5$ (red × markers, along the top of the parallelogram). Case 2 is illustrated by the two sides of the parallelogram with slope approximately 4, where $m = 1$ and $s^2 = 1, 2, 3, 4, 5$ (square yellow markers, along the left side of the parallelogram) and $m = 5$ and $s^2 = 1, 2, 3, 4, 5$ (diamond purple markers, along the right side of the parallelogram). Case 3 is illustrated by the diagonal of the parallelogram with slope approximately 8/3, where $m = s^2 = 1, 2, 3, 4, 5$ (o green markers).

Mean (M) or variance (V) of multiplicative factors $A(t)$	Period	Point estimate	2.5%-tile	97.5%-tile
M	1954-2014	1.03	0.97	1.10
V	1954-2014	0.068	0.045	0.13
M	1977-2014	1.04	0.96	1.14
V	1977-2014	0.079	0.037	0.13

Supplementary Table 1 | Estimates of the parameters from LC theory and their 95% confidence intervals over the periods 1954-2014 and 1977-2014. The confidence intervals are based on 10,000 bootstrap samples.

Supplementary Discussion

Here we give one possible interpretation of our finding that the mean and variance of the number of tornadoes per outbreak approximately satisfy TL with $b = 4.3 \pm 0.44$, i.e., b does not differ significantly from 4. This behavior contrasts with that of the Poisson and negative binomial distributions where the variance is a linear and quadratic function of the mean, respectively. We shall show that $b \approx 4$ is a consequence, under certain conditions which we shall specify, of the multiplicative population process proposed by Supplementary Ref. 2. The lognormal distribution is a limiting distribution of such processes.

By way of background, we recall that a positive-valued random variable X is lognormally distributed if $\log X$ is normally distributed. Supplementary Reference 3 gives an informal introduction to the lognormal distribution. Since the normal distribution is fully specified by its mean m and its variance s^2, the lognormal distribution is fully specified by the same m and s^2. Explicitly, $X(m,s^2)$ is lognormally distributed with parameters m and s^2 if and only if $\log(X(m,s^2))$ is normally distributed with mean m and variance s^2.

When the independent multiplicative factors $A(t)$ have finite mean M and finite variance V, their cumulative product $N(t)$ becomes lognormally distributed as t becomes large[2]. This convergence in distribution is a direct consequence of the central limit theorem applied to $\log N(t+1) = \log N(0) + \log A(0) + \ldots + \log A(t)$, which follows from the definition of $A(t)$ in equation (2) of our main text.

The following facts are standard:

$$\mathrm{E}(X(m,s^2)) \equiv \mu(m,s^2) = \exp(m+s^2/2), \tag{1}$$

$$\mathrm{SD}(X(m,s^2)) = (\mathrm{Var}(X(m,s^2)))^{1/2} \equiv \sigma(m,s^2) = \exp(m+s^2/2)[\exp(s^2)-1]^{1/2}\,, \tag{2}$$

$$\mathrm{CV}(m,s^2) \equiv (\mathrm{Var}(\mathrm{X}(m,s^2)))^{1/2}/\mathrm{E}(\mathrm{X}(m,s^2)) = [\exp(s^2)-1]^{1/2}\,, \tag{3}$$

which is independent of m.

A less-widely known but obvious relationship that is relevant to TL is that

$$\sigma^2(m,s^2) = \mu(m,s^2)^2[\exp(s^2)-1]. \tag{4}$$

Three cases of this relationship are illustrated in Supplementary Fig. 11.

Case 1. If m is changing and s^2 is fixed, $b = 2$. Taylor's law holds exactly.

Case 2. If m is fixed and s^2 is changing, then the variance is not exactly a power law function of the mean because the coefficient $[\exp(s^2)-1]$ is changing. As s^2 increases, both the mean $\mu(m,s^2)$ and the variance $\sigma^2(m,s^2)$ of $X(m,s^2)$ increase. In this case, we can write the variance as a function of the mean since $\mu(m,s^2)^2 = \exp(2m)\exp(s^2)$ and $[\mu(m,s^2)\exp(-m)]^2 = \exp(s^2)$, so

$$\exp(s^2) - 1 = [\mu(m,s^2)\exp(-m)]^2 - 1, \tag{5}$$

and

$$\sigma^2(m,s^2) = \mu(m,s^2)^2[\exp(s^2)-1] = \mu(m,s^2)^2\{[\mu(m,s^2)\exp(-m)]^2 - 1\}. \tag{6}$$

Here the leading term is proportional to $\mu(m,s^2)^4$. For different values of s^2, Taylor's law holds approximately with exponent 4.

Case 3. Suppose that when m changes, s^2 also changes according to $s^2 = m$. In this case,

$$\mu(m,m) = \exp(m+m/2) = \exp(3m/2), \tag{7}$$

$$\sigma(m,m) = \exp(3m/2)[\exp(m)-1]^{1/2} \tag{8}$$

and $\sigma^2(m,m) = \mu(m,m)^2\ [\mu(m,m)^{2/3} - 1]$. In this case the leading term is equal to $\mu(m,m)^{8/3}$. Taylor's law holds not exactly but approximately and the exponent is $2 + 2/3$.

Why would s vary? In an ecological context, a spatial landscape could be composed of patches with varying degrees of disturbance. Highly disturbed patches would be expected to have higher s than less disturbed patches. In a meteorological context, different years might experience different levels of climate variability, e.g., La Nina events or fluctuations in jet streams. Highly disturbed years would be expected to have higher s than less disturbed years.

We suggest that our finding that $b \approx 4$ could arise because, as time is passing, m is relatively steady but s^2 is increasing.

Supplementary References

[1] Cohen, J. E., Xu, M. & Schuster, W. S. F. Stochastic multiplicative population growth predicts and interprets Taylor's power law of fluctuation scaling. *Proc. R. Soc. B,* **280,** 20122955 (2013).

[2] Lewontin, R. C. & Cohen, D. On population growth in a randomly varying environment. *Proc. Natl. Acad. Sci.* (*USA)*, **62,** 1056-1060 (1969).

[3] Limpert, E., Stahel, W., & Abbt, M. Log-normal distributions across the sciences: Keys and clues. *BioScience*, **51,** 341–352 (2001).
